# *Stylobates birtlesi* sp. n., a new species of carcinoecium-forming sea anemone (Cnidaria, Actiniaria, Actiniidae) from eastern Australia

**DOI:** 10.3897/zookeys.89.825

**Published:** 2011-04-11

**Authors:** Andrea L. Crowther, Daphne G. Fautin, Carden C. Wallace

**Affiliations:** 1 *Department of Ecology and Evolutionary Biology, and Natural History Museum and Biodiversity Institute, University of Kansas, 1200 Sunnyside Ave., Lawrence, Kansas 66045–7534 USA*; 2 *Museum of Tropical Queensland, 70–102 Flinders St., Townsville, Queensland 4810 Australia*

**Keywords:** Anthozoa, Hexacorallia, deep sea, symbiosis, hermit crab

## Abstract

We describe a new species of carcinoecium-forming sea anemone, Stylobates birtlesi **sp. n.**, from sites 590–964 m deep in the Coral Sea, off the coast of Queensland, Australia. An anemone of this genus settles on a gastropod shell inhabited by a hermit crab, then covers and extends the shell to produce a chitinous structure termed a carcinoecium. Stylobates birtlesi **sp. n.** is symbiotic with the hermit crab Sympagurus trispinosus (Balss, 1911). The nature of marginal sphincter muscle and nematocyst size and distribution distinguish Stylobates birtlesi **sp. n.** from other species in the genus. The four known species of Stylobates are allopatric, each inhabiting a separate ocean basin of the Indo-West Pacific. We also extend the known range of Stylobates loisetteae in the Indian Ocean off the coast of Western Australia.

## Introduction

Stylobates Dall, 1903, of family Actiniidae Rafinesque, 1815, is an exclusively deep-water genus of sea anemones in which three species are known: Stylobates aeneus Dall, 1903, from Hawai’i and Guam in the Pacific Ocean, Stylobates cancrisocia (Carlgren, 1928a), from the Indian Ocean off Africa, and Stylobates loisetteae Fautin, 1987, from the Indian Ocean off Western Australia. We describe Stylobates birtlesi sp. n. from specimens collected in the Coral Sea off the northeastern coast of Australia. In addition, we report previously unpublished localities for Stylobates loisetteae.

A distinctive feature of Stylobates is the chitinous carcinoecium it produces; a carcinoecium is a shell-like structure inhabited by a hermit crab. Carcinoecia are produced by bryozoans (e.g. Vermeij 1993) as well as many species of cnidarians (e.g. Williams and McDermott 2004), including hydrozoans (e.g. Millard 1975), zoanthids (e.g. Muirhead et al. 1986, Ates 2003) and sea anemones (e.g. Carlgren 1928a, b, Ross 1971, 1984, Dunn and Liberman 1983, Daly et al. 2004). Of anemones that attach to gastropod shells inhabited by hermit crabs, animals of some species (e.g. those belonging to Calliactis) form a thin layer of chitin over the shell. Those of other species (e.g. those belonging to Stylobates and *Paracalliactis*) not only cover but extend the shell, producing a carcinoecium. The ability to form a carcinoecium is a convergent attribute of anemones (Gusmão and Daly 2010); anemones possessing this ability belong to four families (Daly et al. 2004).

The genus and species Stylobates aeneus were described by Dall (1903, p. 61), who was initially under the impression that the shell of “flexible, horny consistency,” which was inhabited by a hermit crab and covered by a sea anemone, was that of a gastropod. He later corrected his mistake, recognizing that “These specimens were secretions from the bases of the Actinias” (Dall 1919, p. 80). Nonetheless, the holotype of Stylobates aeneus Dall, 1903, consisting only of the carcinoecium, is still housed in the mollusc collection of the United States National Museum of Natural History (USNM). Carlgren (1928a) described Isadamsia cancrisocia as a new genus and species of carcinoecium-forming anemone, making no reference to Dall (1903). Dunn et al. (1981) synonymized the genera Isadamsia and Stylobates. According to International Code of Zoological Nomenclature Article 12.2.8 (International Commission on Zoological Nomenclature 1999), having been published before 1931, the name Stylobates aeneus is available for the anemone because it is a “description of the work of an organism.”

The allopatric distribution that we found for the four species of Stylobates is similar to that of other deep-sea invertebrates in the tropical Indo-West Pacific, such as scleractinian corals and squat lobsters.

## Methods

**Figure 1.**
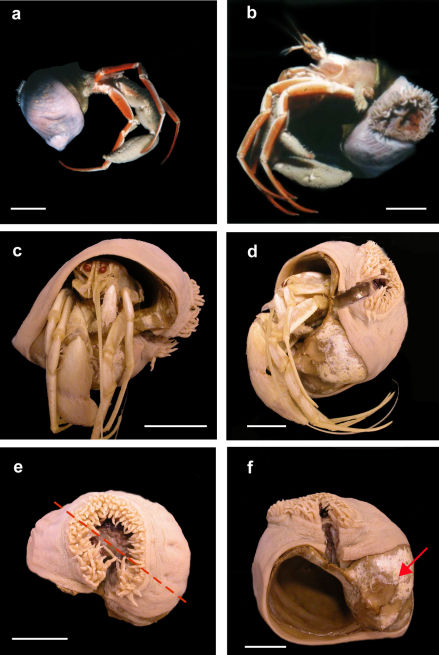
Stylobates birtlesi sp. n. holotype MTQ G57579 **a, b** soon after collection (photo: RA Birtles) **c, d** preserved specimen with Sympagurus trispinosus showing position of oral disc of anemone **e** preserved specimen: shortest tentacles beside longest ones (on right side of oral disc in this view); tentacles grade in length between longest and shortest around other side of oral disc (dashed line indicates directive axis) **f** preserved specimen without hermit crab showing aperture and part of carcinoecium not covered by anemone (arrow). Scale bars 20 mm.

The holotype and five paratypes of Stylobates birtlesi sp. n. were trawled by ORV Franklin during the Cidaris I expedition on the northeastern continental slope of Queensland in 1986 (Anonymous 1986). The holotype was photographed live (Figure 1a, b) within a few minutes of being brought on deck. Four paratypes were trawled by RV *Soela* off the northeast coast of Queensland and one voucher was trawled by FRV *Iron Summer* off the southeast coast of Queensland. All specimens were preserved in 70% ethanol. Specimens of Stylobates loisettae were collected on the RV *Southern Surveyor* expedition to the northwestern coast of Australia in 2007.

Cnida preparations were made from the tentacles, mesenterial filaments, actinopharynx, and column by smashing tissue with water under a coverslip. Preparations were examined using differential interference (Nomarski) optics at 1000×. For each tissue type, the length and width were measured for each type of cnida. Representative cnidae were photographed using an Olympus digital camera. Histological sections were stained with Gomori trichrome (Menzies 1959).

The holotype, four paratype lots, and one voucher of Stylobates birtlesi sp. n. are deposited at Museum of Tropical Queensland, Townsville [MTQ], and one paratype lot is deposited at the Division of Invertebrate Zoology collection of the University of Kansas Biodiversity Institute, Lawrence [KUDIZ]. New records for Stylobates loisetteae are based on specimens in the Western Australian Museum, Perth [WAM]. Separated hermit crab specimens are deposited at Queensland Museum South Bank, Brisbane [QM].

## Results

**Family Actiniidae Rafinesque, 1815**

### Genus Stylobates

Dall, 1903

Stylobates  Dall, 1903: p. 62
Isadamsia  Carlgren, 1928a: p. 167

#### General

Because Dall (1903) had been under the impression that the carcinoecium upon which he based his description was that of a gastropod, the first description of the anemone was by Carlgren (1928a) for Isadamsia cancrisocia from the east coast of Africa. Carlgren (1928a, 1949) is the only person to have defined the genus, and his definition was based on the single species he knew. We update the definition of Stylobates to incorporate information from all four known species.

Deep-sea Actiniidae with very wide pedal disc that covers a gastropod shell inhabited by a hermit crab. Anemone pedal disc secretes carcinoecium. Column smooth, thin-walled. Marginal sphincter muscle endodermal, circumscribed, palmate or pinnate. Tentacles hexamerously arranged; fewer than mesenteries at base. Longitudinal muscles of tentacles and radial muscles of oral disc ectodermal. Mesenteries of 5–6 orders; those of lowest orders complete and sterile, those of highest orders incomplete and fertile. Retractor muscles weak, diffuse; parietobasilar and basilar muscles distinct.

#### Type species (by monotypy):

Stylobates aeneus Dall, 1903.

### Species description

#### Stylobates birtlesi sp. n.

urn:lsid:zoobank.org:act:6B1BF135-854D-4495-BC5D-0D5FAE79EF7D

Figures 1 [Fig F2] [Fig F3] [Fig F4] 5 

##### Material examined.

**Holotype:**

MTQ G57579 (one specimen) (Figure 1).

Type locality: 17°45.99'S, 148°39.09'E, 958–964 m; Coral Sea, off Tully, Queensland, Australia (FRV Franklin, Cidaris I expedition, Station 15–4). Bottom temperature 5.5°C, rocks/mud sediment. Collected 9 May 1986, by RA Birtles and P Arnold. Hermit crab present.

**Paratypes:**

MTQ G57580 (one specimen).

Locality: 17°52'S, 147°08'E, 680–740 m; Coral Sea, off Tully, Queensland, Australia (FRV Franklin, Cidaris I expedition, Station 48–3). Bottom temperature 8.2°C, mud sediment. Collected 17 May 1986, by RA Birtles and P Arnold. Hermit crab separated from carcinoecium, registered as QM W16502 (crustacean collection).

MTQ G57581 (two specimens).

Locality: 17°51.71'S, 147°09.93'E, 881–920 m; Coral Sea, off Tully, Queensland, Australia (FRV Franklin, Cidaris I expedition, Station 49–3). Bottom temperature 6.1°C, rocks/shell debris/sticky mud sediment. Collected 17 May 1986, by RA Birtles and P Arnold.

MTQ G57582 (one specimen).

Locality: 18°01.69'S, 147°20.53'E, 899–918 m; Coral Sea, off Tully, Queensland, Australia (FRV Franklin, Cidaris I expedition, Station 50–3). Bottom temperature 6.2°C, mud sediment. Collected 17 May 1986, by RA Birtles and P Arnold. Hermit crab separated from carcinoecium, registered as QM W16499 (crustacean collection).

KUDIZ 003352 (one specimen).

Locality: 18°01.69'S, 147°20.53'E, 899–918 m; Coral Sea, off Tully, Queensland, Australia (FRV Franklin, Cidaris I expedition, Station 50–3). Bottom temperature 6.2°C, mud sediment. Collected 17 May 1986, by RA Birtles and P Arnold. Hermit crab separated from carcinoecium, registered as QM W16499 (crustacean collection).

MTQ G64680 (four specimens).

Locality: 16°55'S, 151°34'E, 880 m; Coral Sea, northeast Queensland, Australia (RV Soela, Station CO685A78). Collected 6 December 1985, by P Davie. Hermit crabs separated from carcinoecia, registered as QM W16514 (crustacean collection).

**Voucher:**

MTQ G58760 (one specimen).

Locality: 27°59.37'S, 154°00.12'E, 590 m; off coast of southeast Queensland, Australia (FRV Iron Summer, Shot 2). Collected 31 March 1983, by R Morton.

##### Description.

###### Base:

Pedal disc concave, attached to carcinoecium. Base of anemone covers most of carcinoecium, except part directly under hermit crab, presumably where hermit crab’s chelipeds frequently contact carcinoecium (arrow, Figure 1f).

###### Column:

Not cylindrical: wraps around gastropod shell so column much longer on one side than on diametrically opposite side. Smallest specimen with shortest side 4 mm, longest side 50 mm. Largest specimen with shortest side 15 mm, longest side 90 mm. Smooth, thin. Fosse shallow. Live specimens light pink, body wall translucent (Figure 1a, b); preserved specimens beige. Mesenterial insertions visible through body wall; white in live specimens (Figure 1a) and preserved specimens.

###### Oral disc:

Oriented toward substrate in life, over umbilicus area (Figure 1c, d). Disc and mouth circular (Figure 1e); disc exposed and mouth agape in all specimens examined. Ectodermal musculature radial.

###### Orientation:

Directive axis in line with spire of shell, parallel to parietal wall of aperture (dotted red line, Figure 1e).

###### Tentacles:

**Figure 2. F2:**
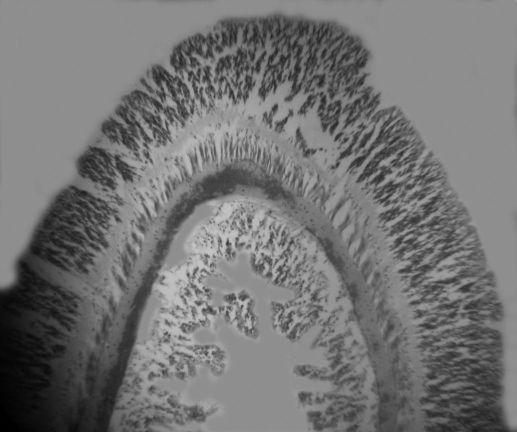
Longitudinal section through tentacle of Stylobates birtlesi sp. n. paratype MTQ G57580.

Beige, slightly darker than column, no pattern. Relatively narrow, tip terete.96 to more than 200 in largest specimens; at margin, in 3 or 4 cycles. Not of uniform length: shortest ones (1–4 mm) on directive axis, at end of one siphonoglyph, beside longest ones (3–9 mm); tentacle length grades between them around oral disc (Figure 1e). Ectodermal musculature longitudinal (Figure 2).

###### Marginal sphincter muscle:

**Figure 3. F3:**
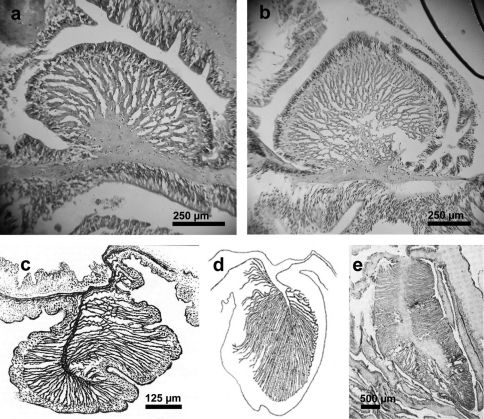
Endodermal circumscribed marginal sphincter muscles of Stylobates spp. **a, b** Palmate marginal sphincter muscle of Stylobates birtlesi sp. n. **a** paratype MTQ G57581 **b** paratype KUDIZ 003352 **c-e** Pinnate marginal sphincter muscles. **c** Stylobates aeneus (from Dunn et al. 1981) **d** Stylobates cancrisocia (from Carlgren 1928a) **e** Stylobates loisetteae (from Fautin 1987).

Well developed, circumscribed, palmate (Figure 3a, b).

###### Mesenteries and internal anatomy:

**Figure 4. F4:**
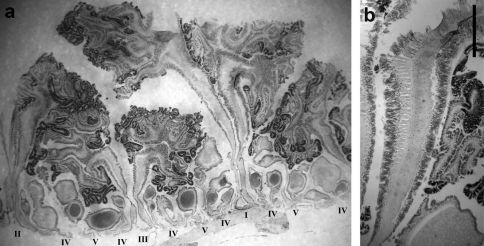
Mesenterial musculature and fertility of Stylobates birtlesi sp. n. holotype MTQ G57579 **a **mesenterial arrangement, orders indicated with Roman numerals; column wall at base of image **b** diffuse retractor muscle; column wall at base of image. Scale bar 1 mm.

Two siphonoglyphs visible in most specimens; actinopharynx ribbed, darker beige than column. Mesenteries to five orders (Figure 4a); thin, each with oral but no marginal stoma. Retractor muscles diffuse (Figure 4b). Parietobasilar muscle with short free penon. Sexes presumably separate: three females, one male examined. First three orders complete and sterile, rest incomplete and fertile (Figure 4a).

###### Carcinoecium:

Shape similar to that of dextral, trochoid gastropod shell. Aperture with simple arced elliptical outer lip, fairly straight parietal wall along what would be termed the columella in a gastropod. Bronze color, becomes chalky out of liquid.

###### Cnidae:

**Figure 5.**
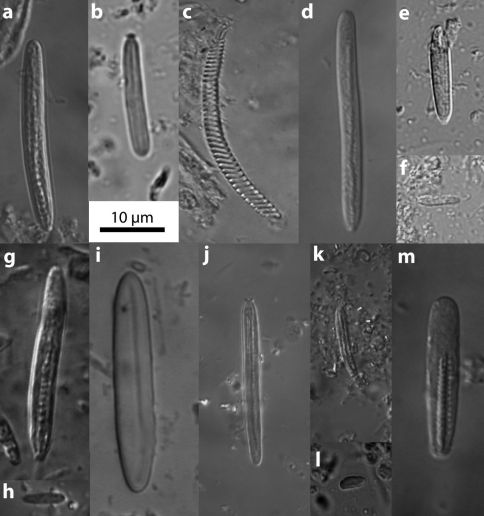
Cnidae of Stylobates birtlesi sp. n. Refer to Table 1 for list of cnida types and distribution.

Cnidom: Spirocysts, basitrichs, microbasic *p*-mastigophores. Table 1 lists distribution and size of cnidae; Figure 5 depicts each cnida type. The largest specimen (MTQ G57580) possessed small basitrichs (*e*)and(*f*) in the actinopharynx, and small basitrichs (*h*) in the column that were not found in other specimens.

**Table 1.** Cnidae of all four species of Stylobates, given as range in length X width of undischarged capsules in µm (outlier measurements in parentheses). n = number of capsules measured, N = ratio of number of animals in which that type of cnida was found to the number of animals examined (where data are available). Frequency of cnida type indicated by the following: ++ very common, + common, - sporadic. Letters in parentheses correspond to images in Figure 5 for Stylobates birtlesi sp. n.

		Stylobates birtlesi sp. n.	Stylobates aeneus	Stylobates cancrisocia	Stylobates loisetteae
Tentacles	Basitrich (*a*)	26.6-38.8 X 2.6-4.6 n=63 N=6/6 [++]	27.9-36.1 X 3.1-3.9 n=43	27.1-30.3 X 2.5-3.3 n=11	29.8-39.7 X 2.5-3.7 n=70 N=6/6
	Basitrich (*b*)	(11.9) 14.2-19.9 X 2.6-3.4 n=12 N=5/6 [ – ]			9.9-16.1 X 1.6-2.5 n=11 N=3/6
	Spirocyst (*c*)	17.9-39.8 (46.3) X 2.2-4.6 n=51 N=7/7 [+]	(25.4) 28.7-44.3 X 2.9-4.1 (4.9) n=40	23.8-39.4 X 2.7-3.3 n=10	21.1-55.8 X 2.5-3.7 n=63 N=6/6
Actinopharynx	Basitrich (*d*)	27.8-37.1 X 2.9-4.3 n=61 N=6/6 [++]	(26.2) 29.5-37.7 X 2.9-4.1 n= 38	27.1-31.2 X 2.5-3.3 n=10	26.0-37.2 X 2.5-3.7 n=59 N=5/5
	Basitrich (*e*)	19.9-21.2 X 3.3 n=9 N=1/6 [ – ]	17.2-21.3 X 2.5-3.1 n=9		
	Basitrich (*f*)	6.4-11.2 X 1.9-2.8 n=20 N=1/6 [ – ]			
Column	Basitrich (*g*)	19.9-30.5 X 2.6-4.1 n=65 N=7/7 [++]	29.5-36.1 X 2.9-3.5 n=24	23.0-28.7 X 2.7-3.3 n=12	21.1-33.5 X 2.5-3.7 n=46 N=5/5
	Basitrich (*h*)	7.3-9.2 X 1.9-3.6 n=10 N=1/6 [ – ]			
Mesenterial Filaments	Basitrich (*i*)	27.5-37.1 X 4.4-6.7 n=25 N=6/6 [++]		(25.4) 27.9-32.8 X 4.1-5.5 n=14	28.5-37.2 X 4.7-6.0 n=34 N=5/7
	Basitrich (*j*)	28.5-33.2 X 2.8-3.8 n=18 N=5/6 [ – ]	29.5-36.1 X 2.5-3.9 n=21		28.5-37.2 X 2.5-3.5 n=11 N=3/7
	Basitrich (*k*)	15.9-21.4 X 2.1-3.1 n=51 N=6/6 [++]	15.6-23.0 X 2.1-3.3 n=58	14.8-18.0 X 2.1-2.5 n=8	12.4-16.1 X 1.9-3.1 n=10 N=4/7
	Basitrich (*l*)	7.9-11.9 X 1.9-2.9 n=21 N=3/6 [+]			
	Microbasic *p*-mastigophore (*m*)	21.2-30.0 X 4.4-7.9 n=45 N=5/6 [++]	21.3-29.5 X 3.9-5.7 n=27	18.9-23.0 X 4.1-5.7 n=12	23.6-32.2 X 3.5-6.2 n=50 N=7/7
Source:		This study	Dunn et al. 1981	Dunn et al. 1981	Fautin 1987

###### Habitat:

Mud and rocks, 590–694 m.

###### Distribution:

**Figure 6.**
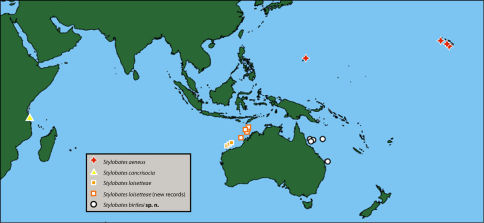
Distribution of species of Stylobates.

From Coral Sea of northern Queensland to southern Queensland coast (Figure 6).

###### Symbiont:

Hermit crab Sympagurus trispinosus (Balss, 1911), identified by Dr. Rafael Lemaitre (Curator of Crustacea, Department of Invertebrate Zoology, USNM).

##### Etymology.

This species is named for R Alastair Birtles of James Cook University, Townsville, who, with the late P Arnold (MTQ) and M Pichon (Australian Institute of Marine Science), collected this species and photographed it alive.

### New records of Stylobates loisetteae Fautin, 1987

WAM Z50046 (one specimen).

Locality: 12.1814°S, 123.4177°E, 400 m; Ashmore, Western Australia, Australia (FRV Southern Surveyor, Station SS0507/189, Beam Trawl). Collected 6 July 2007, by MP Salotti.

WAM Z50047 (one specimen).

Locality: 13.2247°S, 123.3957°E, 400 m; Kulumburu, Western Australia, Australia (FRV Southern Surveyor, Station SS0507/176, Sherman Sled). Collected 5 July 2007, by MP Salotti.

WAM Z50049 (one specimen).

Locality: 13.2650°S, 123.3741°E, 400 m; Kulumburu, Western Australia, Australia (FRV Southern Surveyor, Station SS0507/180, Beam Trawl). Collected 6 July 2007, by MP Salotti.

WAM Z50050 (two specimens).

Locality: 15.6102°S, 120.8076°E, 400 m; Lacepede, Western Australia, Australia (FRV Southern Surveyor, Station SS0507/130, Beam Trawl). Collected 1 July 2007, by MP Salotti.

WAM Z50058 (two specimens).

Locality: 12.5295°S, 123.4273°E, 400 m; Ashmore, Western Australia, Australia (FRV Southern Surveyor, Station SS0507/192, Beam Trawl). Collected 6 July 2007, by MP Salotti.

### Differential diagnosis

Tables 1 and 2 present the major attributes of the four known species of Stylobates. Stylobates birtlesi sp. n. differs from the others in size and distribution of some of its nematocysts (Table 1), and the nature of the marginal sphincter muscle (Figure 3a, b). Compared to Stylobates birtlesi sp. n., Stylobates aeneus and Stylobates cancrisocia do not possess basitrichs (*b*) in the tentacles nor basitrichs (*l*) in the mesenterial filaments; Stylobates aeneus lacks basitrichs (*i*) and Stylobates cancrisocia lacks basitrichs (*j*) in the mesenterial filaments. The clearest distinguishing feature of Stylobates birtlesi sp. n. is the possession of an endodermal circumscribed marginal sphincter muscle in which the lamellae are arranged in a palmate fashion (Figure 3a, b). This is clearly different to the other three species, which all possess an endodermal circumscribed marginal sphincter muscle in which the lamellae are arranged in a pinnate fashion (Figure 3c–e).

**Table 2.** Morphological, biogeographic, and ecological attributes of all four species of Stylobates.

	Stylobates birtlesi sp. n.	Stylobates aeneus	Stylobates cancrisocia	Stylobates loisetteae
*Marginal sphincter muscle*	endodermal, circumscribed, palmate	endodermal, circumscribed, pinnate	endodermal, circumscribed, pinnate	endodermal, circumscribed, pinnate
*Tentacle lengths*	differ around oral disc	differ aroundoral disc	differ around oral disc	marginal greater than oral
*Maximum oral disc diameter (mm)*	15-40	~20	15-30	to 55
*Locality*	NE Australia	Guam and Hawai’i	E Africa	NW Australia
*Depth (m)*	590–964	402–797	818	320–508
*Substrate*	mud, rock	sand	not recorded	mud
*Hermit crab symbiont*	Sympagurus trispinosus	Sympagurus dofleini	Sympagurus trispinosus	Sympagurus brevipes

Gross morphology of Stylobates birtlesisp. n. is similar to that of Stylobates aeneus and Stylobates cancrisocia in position and size of oral disc, and size and arrangement of tentacles. Tentacles of Stylobates birtlesi sp. n. (maximum length 9 mm) are shorter than those of Stylobates loisetteae (maximum length 20 mm). The tentacles of Stylobates loisetteae, in contrast to those of other species, are more or less the same length around the oral disc, and the marginal tentacles are longer than the discal ones. The tentacles of Stylobates aeneus and Stylobates cancrisocia are arranged like those of Stylobates birtlesi sp. n.**,** the longest and shortest ones beside each other (Figure 1e). Diameter of the oral disc of Stylobates birtlesi sp. n. (15-40 mm) is similar to that of Stylobates aeneus and Stylobates cancrisocia,but less than that of Stylobates loisetteae (to 55 mm). The position of the oral disc of Stylobates birtlesi sp. n. is near the aperture of carcinoecium, like in Stylobates aeneus and Stylobates cancrisocia, whereas that of Stylobates loisetteae is on the side of the ultimate whorl of the carcinoecium, away from the aperture.

## Discussion

The four species of Stylobates are distributed allopatrically (Figure 6), in what Cairns (2007) identified as separate biogeographical regions based on distributions of deep-water scleractinian corals. Stylobates birtlesi sp. n. occurs in the Coral Sea off the Queensland coast of Australia (southwestern Pacific region); Stylobates aeneus is known from Hawai’i and Guam (central Pacific region); Stylobates loisetteae occurs in the Indian Ocean off the northwest coast of Australia (southeastern Indian Ocean region); and Stylobates cancrisocia is known from the Indian Ocean off east Africa (southwestern Indian Ocean region). Congeneric species of squat lobsters of the genus Paramunida have a similar allopatric distribution in the central and the southwestern regions of the Pacific (Baba et al. 2008).

Uchida and Soyama (2001) reported Isadamsia sp. J from Japan; that locality is consistent with the distribution of Stylobates aeneus. Doumenc (1975) reported Isadamsia cancrisocia in the North Atlantic at 3360–3600 m. We are dubious about this identification (and do not include the record in Figure 6) because all records for the occurrence of Stylobates are from the Indo-West Pacific and at shallower depths.

Carcinoecium-forming anemones of genera characterized by a mesogleal sphincter muscle are known from the Atlantic: for example, Paracalliactis consors (Verrill, 1882) occurs off the northeast coast of the United States at depths of 2085–2665 m, and Adamsia obvolva Daly et al., 2004, occurs in the Gulf of Mexico at depths of 405–719 m. A specimen of an anemone symbiotic with a hermit crab in the Invertebrate Zoology collection of the California Academy of Sciences (catalog number 35119)from 2630–2660 m off the Pacific coast of Mexico is not Stylobates, as it is labelled, based on its mesogleal sphincter muscle.

A specimen in the Invertebrate Zoology collection of WAM (catalog number Z31227) of an anemone that laid down some chitinous material on the gastropod shell to which it is attached is from the same region and depth as Stylobates loisetteae off the coast of Western Australia,andhas an endodermal sphincter, but does not belong to Stylobates, either. This anemone differs from Stylobates in that its pedal disc does not cover the whole shell; the chitinous material does not form a carcinoecium; the column is more or less cylindrical and is much thicker than that of Stylobates; and the contracted oral disc creates a collar at the margin.

Hermit crabs form symbioses with about 100 species of cnidarians (Williams and McDermott 2004). They occur shallow and deep, in tropical and temperate seas. The hermit crab is thought to be protected by its cnidarian symbiont (e.g. Ross 1971, McLean and Mariscal 1973, Bach and Herrnkind 1980, Brooks 1988, 1989, Brooks and Gwaltney 1993); possible benefits to the cnidarian include transport (Balss 1924, Ross 1974), a firm substrate for attachment (Brooks and Mariscal 1986), and access to food collected by the hermit crab (Ross 1984).

Many carcineocium-forming species occur in the deep sea, where calcium carbonate (the mineral of mollusc shells) is highly soluble (Correns 1955), resulting in a limited supply of shells (Balss 1924) and rarity of large shells. A hermit crab living in a chitinous carcinoecium need not change shells as it grows, nor will the carcinoecium dissolve (Dunn et al. 1981). In symbioses not involving a carcinoecium, more than one anemone may be attached to a gastropod shell inhabited by a hermit crab, whereas in the Stylobates/Sympagurus system, one anemone is associated with one hermit crab. In the most thorough account of this association, Dunn et al.(1981) considered it to be obligate for the anemone (which receives food, transport, and substrate) and facultative for the hermit crab.

Each species of Stylobates is associated with hermit crabs of one species, all belonging to Sympagurus. Stylobates aeneus occurs with Sympagurus dofleini (Balss, 1912), Stylobates cancrisocia and Stylobates birtlesi sp. n. both occur with Sympagurus trispinosus (Balss, 1911) and the hermit crab associated with the newly recorded specimens from the Southern Surveyor cruise of Stylobates loisetteae occur with Sympagurus brevipes (de Saint Laurent, 1972) (A McCallum, Museum Victoria, pers. comm.); hermit crabs of this species are “frequently associated with actinian-secreted carcinoecium similar to that of Stylobates” (McLaughlin et al. 2007, p. 299).

## Conclusion

We describe Stylobates birtlesi sp. n., a new species of deep-sea anemone associated with the hermit crab Sympagurus trispinosus (Balss, 1911), from specimens collected in the Coral Sea off the Queensland coast of Australia. Stylobates birtlesi sp. n. differs from the other three known species of Stylobates in some aspects of its nematocysts, and in having a palmate marginal sphincter muscle (in the others it is pinnate). The four species of Stylobates are allopatrically distributed in the deep Indo-West Pacific Ocean, a pattern similar to those of deep-sea scleractinian corals and squat lobsters.

## Supplementary Material

XML Treatment for Stylobates

XML Treatment for Stylobates birtlesi
